# Diurnal Hypothalamic-Pituitary-Adrenal Axis Measures and Inflammatory Marker Correlates in Major Depressive Disorder

**DOI:** 10.3390/ijms18102226

**Published:** 2017-10-24

**Authors:** Kelly Doolin, Chloe Farrell, Leonardo Tozzi, Andrew Harkin, Thomas Frodl, Veronica O’Keane

**Affiliations:** 1 Trinity College Institute of Neuroscience, Dublin 2, Ireland; farrelc6@tcd.ie (C.F.); leo.tozzi88@gmail.com (L.T.); aharkin@tcd.ie (A.H.); vokeane@tcd.ie (V.O.); 2 Department of Psychiatry, Otto von Guerick University, 39106 Magdeburg, Germany; thomas.frodl@med.ovgu.de; 3 Trinity Centre for Health Sciences, Tallaght Hospital, Tallaght, Dublin 24, Ireland

**Keywords:** major depressive disorder, hypothalamic-pituitary-adrenal axis, immune system, cortisol awakening response, inflammation

## Abstract

Dysregulation of the hypothalamic-pituitary-adrenal (HPA) axis and inflammatory systems is a consistent finding in patients with Major Depressive Disorder (MDD). Cortisol is often assessed by measurement of the cortisol awakening response (CAR) and/or diurnal cortisol levels. Some methods of cortisol measurement overestimate cortisol concentration due to detection of other glucocorticoids including the relatively inert cortisone, therefore this study aimed to assess the presence of both cortisol and cortisone, and the cortisol-cortisone catalyzing enzyme 11β-hydroxysteroiddehydrogenase type 1 (11β-HSD1), in depressed patients and controls. Because the HPA axis is known to regulate the body’s immune system, relationships between measures of cytokines and cortisol were also assessed. Saliva samples were collected from 57 MDD patients and 40 healthy controls at five post-wakening time points (0, +30, +60, +720 and +750 min). Glucocorticoid concentrations were measured by liquid chromatography mass spectrometry. Whole blood mRNA expression of several inflammatory markers was measured by quantitative polymerase chain reaction. This study replicated the common finding of elevated morning cortisol and reduced CAR reactivity in MDD and found no differences in cortisone or 11β-HSD1 mRNA measures. There was a negative association between interleukin 1-β (IL-1β) mRNA and morning cortisol reactivity within the depressed group, indicating that dysregulation of the HPA axis and immune system may be interconnected.

## 1. Introduction

Major Depressive Disorder (MDD) is a widespread psychiatric disorder that has been identified as the leading cause of suicide [1] and has a high lifetime prevalence rate of 16% [2]. Despite its prevalence, the biological etiology of depression remains elusive. Stress has been shown to be a major risk factor in developing depression [3] and further investigation of biological pathways relating to stress in a depressed population may help to understand the stress-related etiology of depression.

The hypothalamic pituitary adrenal (HPA) axis is a major part of the neuroendocrine system that controls stress responses to the environment. Hyperactivity of the HPA axis, exhibited as elevated cortisol concentrations, is one of the most consistent findings in the search for biological etiology in MDD [4]. It has been hypothesized that this hyperactivity is due to reduced efficacy of central glucocorticoid receptor function that results in dysfunctional HPA axis feedback [5,6]. One measure commonly used to assess HPA axis activation is the cortisol awakening responses (CAR) [7], i.e., the rapid increase in cortisol secretion roughly within the first 30 min of wakening that occurs daily, signifying the physiological stress response to waking [8]. Past studies have found elevated wakening cortisol concentrations and blunted awakening responses in MDD [9,10,11].

In some methods of cortisol measurement, such as enzyme linked immunosorbent assays (ELISA), cortisol concentrations are inadvertently overestimated due to erroneous detection of glucocorticoids including cortisone. Cortisone is a relatively inert glucocorticoid due to its poor binding to the glucocorticoid and mineralocorticoid receptors, therefore its presence does not necessarily indicate recruitment of an immune response in the way that hypercortisolemia is associated with a lack of suppression of inflammatory responses. However, cortisone can be readily enzymatically converted inside cells to the active steroid cortisol by the enzyme 11β-HSD1 [12]. Cortisone injections are used as treatment to suppress inflammatory conditions such as arthritis, as cortisone is made available to convert to cortisol in peripheral tissue. Injections of cortisone for treatment of inflammatory conditions have the known side effect of increasing anxiety and depression [13], indicating that it too may play a role in the pathophysiology of depression. 

11β-HSD1 has been implicated in human HPA axis regulation and susceptibility to depression. Subjects with the rs11119328 polymorphism of the gene that encodes for 11β-HSD1, *HSD11β-1*, were found to have higher cortisol levels and increased rates of depression [14]. Furthermore, ablation of the gene that encodes for 11β-HSD1 in mice results in anti-depressant effects during the forced swim test [15]. Also, 11β-HSD1 has been linked to inflammatory conditions [12]. These findings indicate that the involvement of the HPA axis in depression may be characterized by more than an increase in cortisol concentration alone, and that cortisone and 11β-HSD1 should be considered in the assessment of HPA axis activity in depressed patients.

The HPA axis is functionally linked to the immune system as glucocorticoids regulate inflammatory responses [16] and increased inflammation is an established consequence of stress system activation [17]. It has been shown that dysregulation of the HPA axis results in lack of suppression of the immune system, known as glucocorticoid resistance [18]. It is possible that these two pathways are both altered in depression as a result of their functional relationship. Activated inflammatory pathways have been repeatedly observed in MDD patients [19], as demonstrated by increased inflammatory cytokines. Most consistently reported is the increase of interleukin (IL)-6 concentrations in circulating serum or plasma of depressed patients [20,21,22]. Increases in plasma IL-1β and tumor necrosis factor (TNF)-α have also been described in depressed patients compared to non-depressed people [20,23,24]. Additionally, severity of depression symptoms has been shown to be correlated to inflammation [24].

The aims of this study were to compare HPA axis activity between depressed patients and healthy controls, with a more specific measure of salivary cortisol and cortisone concentrations using the liquid chromatography-mass spectrometry (LC-MS) technique. Whole blood mRNA expression of *HSD11β-1* was also measured to investigate its contribution to possible differences in glucocorticoid concentrations. Lastly, whole blood mRNA expression of several inflammatory cytokines was measured to determine the relationship between HPA axis dysregulation and inflammation within a depressed cohort.

## 2. Results

### 2.1. Demographics and Psychiatric Measurements

There were no differences in age, gender, body mass index (BMI), or smoking between healthy controls and patients with MDD recruited to this study. Depressed participants were found to have significantly lower duration of education as per the International Standard Classification of Education (ISCED) scale (*p* < 0.001) compared to healthy controls (HC) (Table 1).

Consistent with a diagnosis of MDD, patients scored significantly higher compared to healthy controls on the Hamilton Depression rating scale (HAM-D) and Center for Epidemiological Studies-Depression scale (CES-D), signifying increased depressive symptoms. Patients also exhibited higher sleep disturbance reflected in elevated Pittsburgh Sleep Quality Index (PSQI) scores. In the depressed group, a greater number of participants had experienced Early Life Adversity (MDD: 61% vs. HC: 21%), with significantly higher global Childhood Trauma Questionnaire (CTQ) scores than healthy controls. Of the depressed cohort, 25 were medication free, 19 were prescribed selective serotonin reuptake inhibitors (SSRIs), 7 were prescribed selective norepinephrine reuptake inhibitors (SNRIs), and 6 were on other medications including benzodiazepines, melatonergics, anti-epileptics or a combination of medications (Table 1).

### 2.2. Salivary Cortisol Concentrations iIn Depressed Patients and Healthy Controls

**Figure 1:**
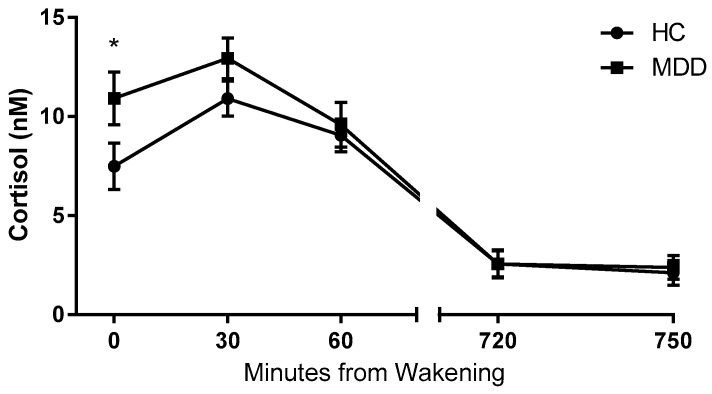
Salivary cortisol concentration data. Salivary cortisol concentrations at five time points from wakening in depressed patients (MDD) (*n* = 57) compared with healthy controls (HC) (*n* = 41). Depressed patients exhibited a statistically significantly higher cortisol concentration at 0 min from wakening. Data are expressed as means and SEM. * *p* < 0.05 vs. control. (Mann Whitney-U test on log transformed data).

A Mann Whitney-U test performed on log transformed cortisol data revealed significantly higher concentrations of cortisol at wakening in MDD patients relative to control subjects (Figure 1). There were no significant difference in cortisol concentration between depressed patients and healthy controls at time points T30, T60, T720 or T750 (Table 2). Analysis revealed no significant differences in the average morning or evening cortisol, or in diurnal variation between groups.

### 2.3. Cortisol Awakening Response in Depressed Patients and Healthy Controls

**Figure 2:**
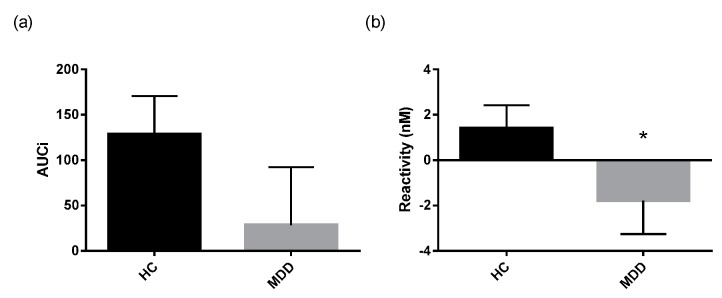
CAR responsivity measures in patients and healthy controls.(**a**) area under the curve with respect to increase(AUCi) and (**b**) cortisol awakening response (CAR) reactivity expressed as mean and SEM in depressed patients (*n* = 57) and healthy controls (HC) (*n* = 41). * *p* ≤ 0.05 vs. control. (Mann Whitney-U test on log transformed data).

**Figure 3:**
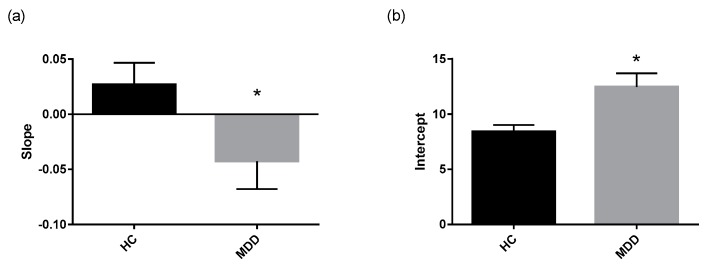
Parameters of regression line fit through morning cortisol data. Regression lines fitted through the morning cortisol data of each of the study group were significantly different. (**a**) The slope of the regression line and (**b**) the intercept of the regression line fitted through morning cortisol concentrations in depressed patients (*n* = 57) compared to controls (*n* = 41) are displayed as means with SEM. * *p* ≤ 0.05 vs. control (Mann Whitney-U test on log transformed data).

The cortisol awakening response (CAR) was assessed in depressed patients and controls using several established measures that describe the Area Under the Curve (AUC) as well as parameters that describe the regression line fitted through the morning cortisol data (Table 3). Mann Whitney-U tests performed on log transformed cortisol data revealed lower reactivity in depressed patients compared to healthy controls (Figure 2b). Additionally, the regression line fitted through the morning cortisol data had a significantly lower slope and higher intercept in depressed patients compared to healthy controls (Figure 3a,b). 

**Figure 4:**
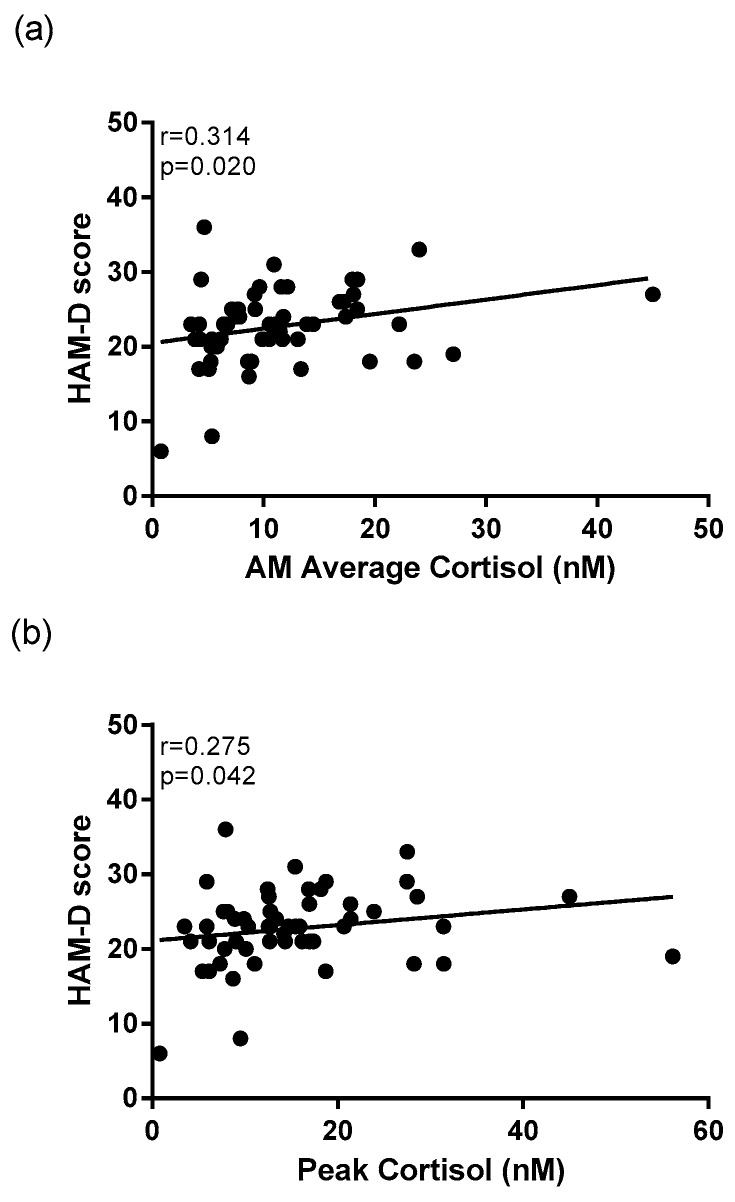
Correlational analysis between depression rating scales and cortisol awakening parameters in MDD patients. A significant positive correlation exists between Hamilton Depression rating scale (HAM-D) scores of depressed patients and (**a**) average morning cortisol concentration, and between (**b**) peak morning cortisol (*n* = 55).

Average morning cortisol concentration (*r* = 0.314, *p* = 0.020) (Figure 4a), peak morning cortisol concentration (*r* = 0.275, *p* = 0.042) (Figure 4b), and cortisol concentration at T30 (*r* = 0.352, *p* = 0.013) were all correlated to HAM-D scores within the depressed group. Correlational analysis revealed that among the entire cohort, there were significant positive relationship between average morning cortisol concentration (*r* = 0.244, *p* = 0.017) and peak morning cortisol concentration (*r* = 0.253, *p* = 0.017) with PSQI scores. 

### 2.4. Salivary Cortisone Concentrations in Depressed Patients and Healthy Controls

There were no differences in salivary cortisone concentrations between depressed patients and healthy controls (Table 4).

### 2.5. Relative Quantification of Whole Blood HSD11β-1 mRNA Expression in Depressed Patients and Healthy Controls

**Figure 5:**
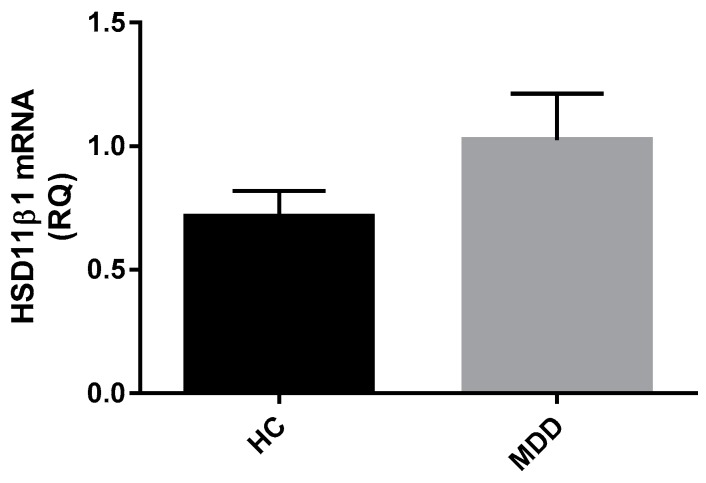
Relative quantification of *HSD11β-1* in MDD patients and healthy controls. Data displayed as means with SEM. There is no difference in gene expression of *HSD11β-1* between MDD patients (*n* = 32) and healthy controls (*n* = 18). (Mann Whitney-U test performed on log transformed RQ data).

Gene expression of *HSD11β-1* was measured in whole blood from a subset of depressed patients (*n* = 42) relative to control subjects (*n* = 21). There were no differences in whole blood *HSD11β-1* mRNA expression between depressed and healthy controls (Z = −0.490, *p* = 0.624) (Figure 5).

**Figure 6:**
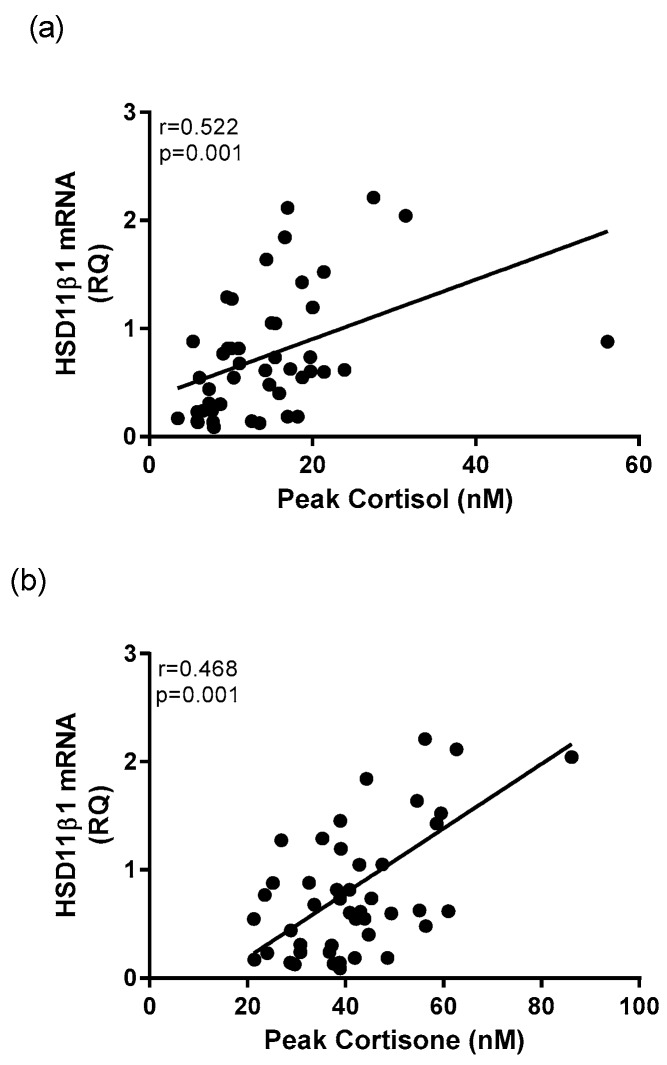
Correlational analysis between whole blood *HSD11β-1* mRNA and glucocorticoid concentrations. A significant positive correlation exists between mRNA expression of *HSD11β-1* and (**a**) peak cortisol concentration and (**b**) peak cortisone concentration.

The relative quantification of whole blood mRNA expression of *HSD11β-1* was significantly correlated to cortisol and cortisone morning concentrations in healthy controls and depressed patients. There was a significant positive relationship between transcriptional expression of *HSD11β-1* and average morning cortisol concentrations (*r* = 0.533, *p* < 0.001) and to peak morning cortisol concentration (*r* = 0.522, *p* < 0.001) in the entire cohort (Figure 6a). Expression of *HSD11β-1* was significantly positively correlated average morning cortisone (*r* = 0.419, *p* = 0.003, and peak cortisone concentration (*r* = 0.468, *p* = 0.001) (Figure 6b).

### 2.6. Whole Blood mRNA Expression of Inflammatory Cytokines

**Figure 7:**
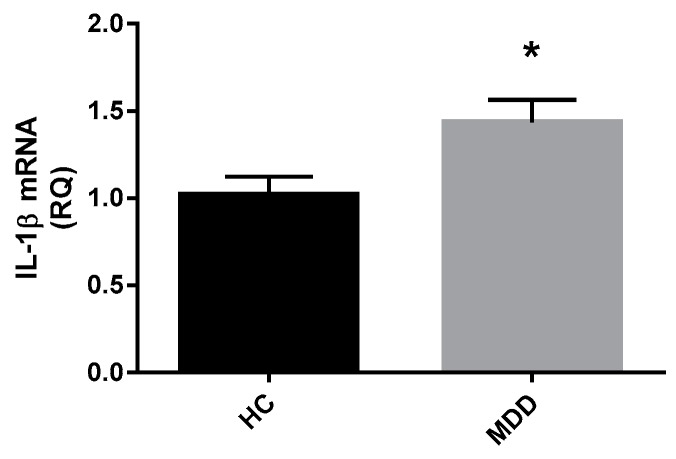
Whole blood mRNA expression of) *IL-1β* in depressed patients and healthy controls.Relative quantification of mRNA expression for *IL-1β* in depressed patients (a: *n* = 42) compared with healthy controls (*n* = 21). Data expressed as mean with SEM. Statistical analysis was conducted with a Mann Whitney-U test on log transformed RQ data. * *p* < 0.05 vs. controls.

Gene expression of *IFN-γ*, *IL-1β*, *IL-6*, and *TNFα* was measured in whole blood from a subset of depressed patients (*n* = 42) relative to control subjects (*n* = 21). A Mann Whitney-U test performed on log transformed relative quantification mRNA expression data revealed significantly higher expression of *IL-1β* mRNA in depressed patients compared to healthy controls (Z = −2.236, *p* = 0.025) (Figure 7). There was no significant difference in *IFN-γ* mRNA expression (Z = −0.620, *p* = 0.535), *IL-6* mRNA expression (Z = −0.232, *p* = 0.817) or *TNFα* mRNA expression (Z = −1.071, *p* = 0.284) between depressed patients and healthy controls.

**Figure 8:**
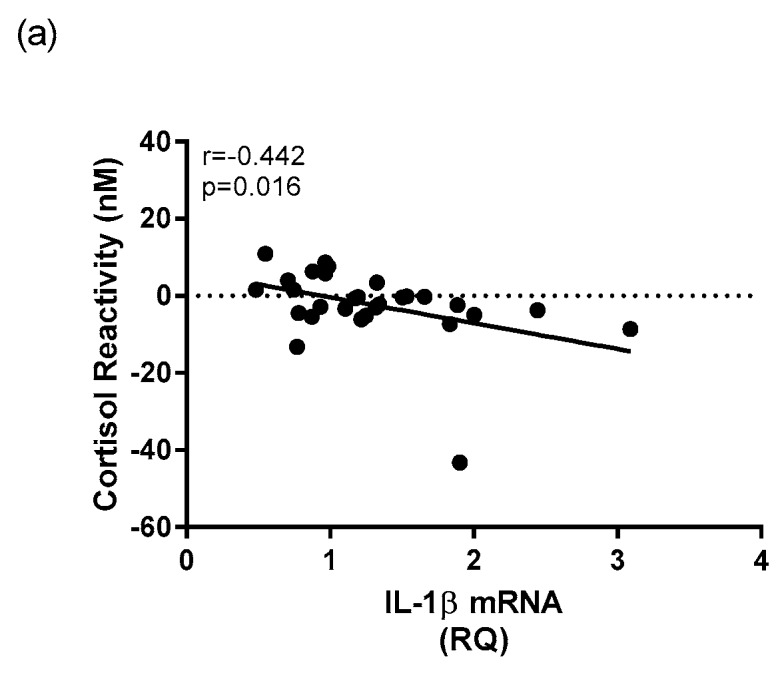

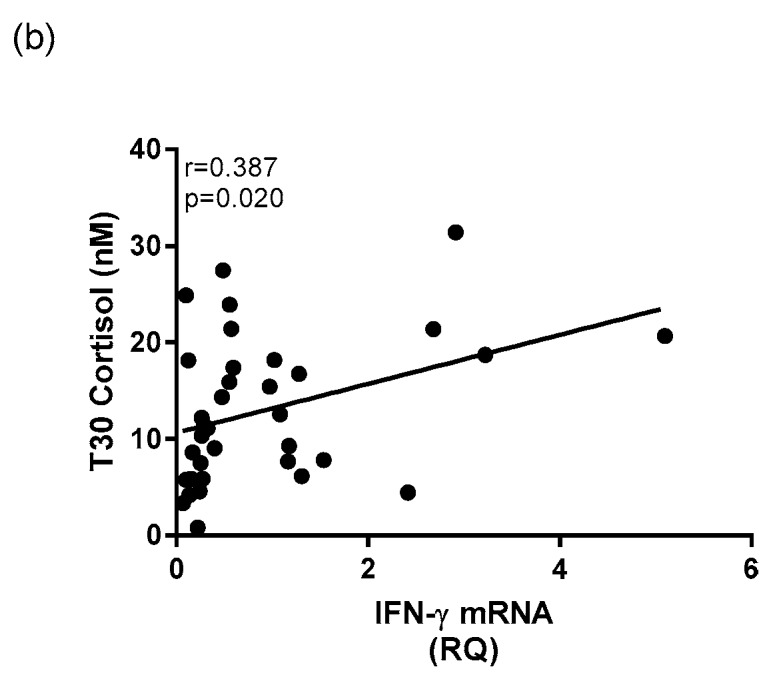
Correlational analysis between mRNA expression of cytokines and HPA axis measures in depressed patients. A Spearman’s rho correlation revealed (**a**) a significant negative correlation between mRNA expression of *IL-1β* and morning cortisol reactivity within the depressed group and (**b**) a significant positive correlation between mRNA expression of *IFN-γ* and T30 cortisol concentration within the depressed group.

Within the depressed group, there was a negative relationship v between whole blood *IL-1β* mRNA expression and cortisol reactivity (*r* = −0.442, *p* = 0.016) (Figure 8a). There was also a significant positive correlation between whole blood *IFN-γ* mRNA expression and cortisol concentration at T30 (*r* = 0.387, *p* = 0.020) within the depressed group (Figure 8b). There were no significant relationships found between mRNA expression of *TNFα* with any HPA axis measures within the depressed cohort.

## 3. Discussion

Overall, the findings from this study showed elevated salivary cortisol concentrations at wakening and reduced CAR reactivity in depressed patients using the LC-MS method of laboratory analysis. There was no difference in salivary cortisone measurements between groups, and no increase in the whole blood mRNA expression of *HSD11β-1*, the enzyme that converts cortisone to cortisol, though expression of this enzyme was significantly related to greater total glucocorticoid output across all participants. The depressed group had elevated levels of whole blood *IL-1β* mRNA, which was also found to be inversely related to CAR reactivity in the depressed group, suggesting a link between dysregulation of the HPA axis and immune system.

### 3.1. Altered Cortisol Awakening Responses in MDD

There are many benefits to using LC-MS for corticosteroid quantification [25]. The specificity of this technique is well documented, and the possibility of measuring multiple analytes simultaneously is an advantage [26,27]. Immunoassays such as ELISA are noted for their high-throughput-ness and their high sensitivity; however they are known to include unspecific binding of molecules with similar structures [28]. When performing the ELISA method, it is not possible to discern what percentage of the detected corticosteroid represents cortisol versus non-specifically bound cortisone, whereas the LC-MS method is capable of measuring concentrations of each independently because of its specificity in identifying these differently functioning molecules [25]. For this reason, LC-MS should be considered a new standard for steroid measurement. 

The results of this study support the hypothesis of HPA axis dysregulation in depressed patients. Wakening cortisol concentration is a commonly used indicator of HPA axis activity and determination of a stress response, as it is representative of the body’s response to the stressful biological event of waking up [7]. In this study, the depressed group displayed significantly higher wakening (T0) salivary cortisol concentrations than healthy controls, representative of a hyper-active stress system. This is a finding that has been reported widely in the literature, and the results from this study are in accordance with the existing evidence that stress is central to the pathophysiology of depression [4,29].

Not only did the depressed group display higher cortisol wakening concentrations, but the CAR dynamics were significantly different than that of the healthy control group. The reactivity measure was lower in depressed patients, indicating a blunted CAR relative to controls. The regression lines fitted through morning cortisol data were significantly different between depressed patients and controls, as the depressed group exhibited a higher intercept and lower slope. This reinforces the observation that depressed patients display higher initial concentrations of cortisol and lower fluctuation of cortisol concentration over the course of the morning.

Both groups’ cortisol levels decreased significantly between the morning and evening time points, with average evening cortisol concentrations falling below 3 nM in each group. This is a typical pattern of diurnal variation in cortisol secretion [30,31]. There were no significant differences in diurnal variation between groups which have been reported in the literature [32]. This could be due to inconsistencies in the timing of saliva sampling, since sampling times were scheduled by their distance from wakening time, rather than an absolute time as has been done in other studies (i.e., 12 and 12.5 h from wakening, rather than at 8 PM).

This study also revealed significant correlations between cortisol activity and depressive symptomatology. HAM-D scores within the depressed group were significantly positively correlated to peak morning cortisol. This supports the hypothesis that HPA axis dysregulation is exacerbated with increased severity of depressive symptoms, which has also been reported in previous studies [11,33].

The results of this study suggest a link between disruption of sleep and the HPA axis. Several significant associations between morning cortisol concentrations and sleep disturbance as measured by the self-rated PSQI were found when correlating scores of the whole cohort. While depressed participants scored significantly higher on the PSQI compared to healthy controls, the range of scores within each group and across the entire cohort was broad. Wakening cortisol concentration, average morning cortisol concentration, and peak morning cortisol were each significantly positively correlated to PSQI scores of the entire cohort. Cortisol activity is known to play an essential role in maintenance of normal circadian rhythms [34]. These findings reiterate the relationship between HPA axis dysregulation and sleep disturbance that has been reported in previous research [35,36]. Previous research indicates that people with poor sleep are at risk of mental health consequences [37,38]. Based on the findings of this study and previous studies, poor sleep could be used in the future to identify an opportunity for early intervention with people who are at risk of developing depression. It is possible that treating sleep disruption of non-depressed patients could be useful in regulating the HPA axis to prevent low mood [39,40].

### 3.2. Cortisone and HSD11β-1

There were no differences in cortisone concentrations between depressed patients and healthy controls at any of the five time points. This indicates that the disruption of the HPA axis witnessed in depressed patients is occurring as an increase of cortisol while maintaining similar levels of cortisone. The enzyme 11β-HSD1 that converts cortisone to cortisol has been implicated in human HPA axis regulation and susceptibility to depression and a certain polymorphism of *HSD11β-1* (rs11119328) has been associated with increased rates of depression [41]. Because of the implications identified in the literature, differences in whole blood *HSD11β-1* mRNA expression were anticipated between depressed patients and healthy controls. Contrary to the aforementioned findings, the present study detected no differences in mRNA expression of *HSD11β-1* between groups. However, several significant correlations were found between morning corticosteroid concentration and transcriptional expression of *HSD11β-1* including peak cortisol and cortisone measures. This finding is in line with previous work that has demonstrated that increased cortisol is associated with increased mRNA expression of *HSD11β-1* [14]. The existing literature exploring the role of 11β-HSD1 in depression is limited but this enzyme may play a part in the regulation of cortisol concentration and serve as a marker of cortisol activity. These findings also suggest that while cortisone and 11β-HSD1 may play a role in HPA axis dysregulation, morning salivary cortisol measurements remain the strongest marker of HPA axis disruptions in MDD.

### 3.3. Inflammatory Markers and Their Relation to HPA Axis Activity

Whole blood transcriptional expression of *IFN-γ*, *IL-1β*, *IL-6*, and *TNF-α* was measured by qPCR. The resultant relative quantification data indicated a significantly higher level of *IL-1β* expression in depressed patients compared to healthy controls, suggesting a mildly altered immune activation in the MDD group. However, the present study did not replicate previous findings that have indicated increased gene expression or involvement of *IL-6*, *IFN-γ*, or *TNF-α* in depression [42,43].

Circulating IL-1β is produced by active macrophages in the periphery as well as by glial cells and neurons in the central nervous system [44]. This interleukin is known as a driver of immune responses [45] and promotes sickness behavior [46]. Knowledge of the role of IL-1β in mood disorders has been established through several means including epidemiological data [47], studies showing the modification of *IL-1β* expression following treatment of mood disorders [48], and the behavioral alterations documented following administration of IL-1β in pre-clinical studies [49]. The findings of this study support the existing literature.

Inflammation is known to be a biological consequence of stress and HPA axis alterations [18]. The current study revealed several significant relationships between measures that indicate HPA axis activity and immune activity. For example, within the depressed group, whole blood mRNA expression of *IL-1β* was significantly negatively correlated to morning cortisol reactivity. Decreased morning cortisol reactivity was a measure found to be associated with depression diagnosis, indicating that decreased CAR reactivity could be a marker of depression. Its relationship within the depressed group to whole blood mRNA expression of *IL-1β* is indicative of a link between HPA and immune dysregulation in depression. Also, increased gene expression of inflammatory cytokine *IFN-γ* was associated with increased cortisol at T30 within the depressed group, giving more support to the theory that increased dysfunction of the HPA axis is related to increased inflammation. These findings are in accordance with previous studies that have found links between HPA axis and immune system activation in tandem, and in depression [50,51,52,53].

Not only does this paper strengthen links between HPA axis activation, inflammation, and MDD, it may offer an explanation for previously observed relationships between known MDD comorbidities including blood pressure irregularities. Increased *IL-1β* gene expression has been shown to be positively associated with heart rate measurements and systolic blood pressure reactivity, which may contribute to the cardiovascular risk in people suffering MDD [54]. Evaluating the involvement of inflammatory pathways in common MDD comorbidities may reveal further mechanistic links explaining why these disease states often coexist.

### 3.4. Limitations

This study had several limitations that could be addressed by future research. For purposes of practicality and compliance, participants in this study were asked to complete only 5 saliva sample collections over the course of the day. An increased number of morning saliva samples and smaller time increments would have strengthened the ability to interpret the CAR. These limitations are discussed at length in a review by Stalder [55]. The study would also have benefited from the addition of record keeping by the participants regarding the actual times of saliva sample collection, instead of times relative to wakening. It has been shown that waking time can affect HPA axis activity [55], therefore this should have been a consideration in the study design. Finally, this study would have benefited from recording other participant lifestyle information such as exercise or phase of menstrual cycle at the time of saliva sample collection, as these factors have been shown to impact salivary cortisol measurements [56,57].

In future studies, it would be of interest to measure whole blood mRNA expression of the gene encoding for the enzyme 11β-hydroxysteroid dehydrogenase Type 2 (*11β-HSD2*) in addition to *11β-HSD1*. While 11β-HSD1 catalyzes the conversion of inert cortisone to active cortisol, 11β-HSD2 catalyzes the opposite reaction. Previous studies have suggested the involvement of 11β-HSD2 in anxiety and depression. One study showed that 11β-HSD2 was significantly negatively correlated to maternal prenatal anxiety in humans [58] while another study showed that a knock-out of the *11β-HSD2* gene in the fetal brain of male mice caused depressive symptoms and cognitive dysfunction as they became adults [59]. To gain a more complete understanding of the role of the isoenzyme in mediating altered glucocorticoid concentrations, both enzymes should be considered.

Another limitation of this study was that patients and controls were young, and most participants were Europeans. Younger people have been shown to have higher heterogeneity of IL-6 [60] and this might lead to non-significant finding in *IL-6* mRNA expression. Ethnic origin played an important role in the relationship between levels of cytokines and MDD [60]. As a result, the findings of this study could not be generalized to non-Europeans.

## 4. Materials and Methods

### 4.1. Recruitment

In this study, 57 patients with Major Depressive Disorder (MDD) and 40 healthy controls under the age of 45 with no other chronic diseases or psychotic disorders were included. Depressed patients were recruited from the psychiatric outpatient clinic at Sheaf House in Tallaght Hospital, Dublin 24 and at the Mary Mercer Healthy Centre in Jobstown, Dublin 24. Recruitment of depressed patients was based on criteria for a Major Depressive Episode [61] and a Hamilton Depression (HAM-D) rating scale score of >17, which was assessed during a clinical interview with a consultant psychiatrist.

All participants gave informed consent. Demographic data including age, gender, body mass index (BMI), smoking (yes/no), and educational achievement level based on the International Standard Classification of Education (ISCED) were collected. Ethical approval was granted for this study by the Tallaght Hospital/St. James’s Hospital Joint Research Ethics Committee (REC Reference: 2014/12/05/2015-03 List 11(1)).

### 4.2. Psychiatric Rating Scales

Psychiatric rating scale data was collected by researchers to measure severity of depressive symptoms, sleep disturbance, and childhood trauma in participants. These scales included the HAM-D, Centre for Epidemiology Scale of Depression (CES-D), Pittsburgh Sleep Quality Index (PSQI), and Childhood Trauma Questionnaire (CTQ).

The 21-question HAM-D has been validated and standardized as a measure of depression, and includes questions that address symptoms and experiences including suicidal ideation, insomnia, anxiety, somatic symptoms, and more [62,63]. The total HAM-D score is calculated as a sum of the first 17 questions, while the remaining four provide supplementary clinical information.

The CES-D scale is a 20-item self-report Likert scale. It is completed by the participant at the time of recruitment to assess the perceived severity of their depression symptoms [64]. Questions refer to symptoms associated with depression, including restless sleep, poor appetite, and feelings of loneliness. Response options range from 0 to 3 for each item (0 = “Rarely or None of the Time”; 1 = “Some or Little of the Time”; 2 = “Moderately or Much of the time”; 3 = “Most or Almost All the Time”). Scores range from 0 to 60, with higher scores indicating greater depressive symptoms.

The PSQI is a questionnaire pertaining to the participant’s sleep habits over the course of the last month [65]. It contains 19 questions that generate seven “component” scores including subjective sleep quality, sleep latency, sleep duration, habitual sleep efficiency, sleep disturbances, use sleep medication, and daytime dysfunction. The sum of the seven “component” scores results in a global score ranging from 0 to 21, for which a higher score indicates lower sleep quality.

The CTQ is a standardized, 28-item self-report instrument that assesses five categories of childhood maltreatment including emotional, physical, and sexual abuse, and emotional and physical neglect [66]. The questionnaire is comprised of five questions for each subscale of childhood maltreatment, in addition to 3 items meant to evaluate minimization and denial to identify participants who might be under reporting traumatic events. The CTQ global scores were calculated using the sum of the five category scores. Participants were classified as being Early Life Adversity (ELA) positive if they had scored above the “moderate abuse” threshold for one or more categories.

### 4.3. Saliva Sample Collection

Saliva samples were collected into Salivette^®^ tubes (Sarstedt, Nümbrecht, Germany) by the study participant at five time points throughout the course of one day. These time points were 0, 30 and 60 min after waking, and 12 and 12.5 h after waking. The completed sets of samples were returned in person or by post within one week of sample collection. Salivette^®^ tubes were centrifuged for 10 min at 3000 rpm and room temperature to extract the saliva from the insert. The saliva was aliquoted and stored in microtubes at −80 °C until analysis.

### 4.4. Cortisol and Cortisone Measurement by Liquid Chromatography-Mass Spectrometry (LC-MS)

Saliva samples were assessed for cortisol and cortisone content by liquid chromatography-mass spectrometry (LC-MS) at the University of Manchester in the Department of Clinical Biochemistry. A Shimadzu Prominence LC system (Shimadzu, Milton Keynes, UK) was used for chromatography. The eluate was injected directly into a Quattro MicroTM tandem mass spectrometer (Waters, Manchester, UK). MassLynx NT 3.5 software was used for system control and data processing. This software used the height of the detected peaks, 1/x weighting and linear least-squares regression to produce a standard curve to derive concentrations.

### 4.5. Cortisol Awakening Response (CAR) Calculations

The cortisol awakening response (CAR) was assessed in depressed patients and healthy controls by calculating Area Under the Curve (AUC), peak, reactivity, and parameters of a regression line fitted through the morning cortisol measurements (T0, T30, T60). These calculations are established measures used to describe total morning free cortisol output and cortisol responsivity [67]. Equations for deriving the CAR parameters are displayed in Table 5. Additionally, cortisol/cortisone ratios were calculated for each of the five timepoints to assess relative glucocorticoid quantities.

### 4.6. Measurement of Whole Blood mRNA Expression of HSD11β-1 and Inflammatory Cytokines by qPCR

PAXgene whole blood mRNA expression of *HSD11β-1, IL-1β, IFN-γ*, and *TNF-α* were assessed by quantitative polymerase chain reaction (qPCR). Collection of 2.5 mL of blood was taken into PAXgene mRNA tubes from each participant at the time of recruitment and were stored as per manufacturer’s instructions. Total RNA was isolated from the whole blood and equalized before it was used for cDNA synthesis. Analysis of gene expression of the target gene was conducted using Real-Time PCR methods and Taqman^®^ Gene Expression Assays (Applied Biosystems, Foster City, CA, USA). At the end of the reaction, data analysis was performed with the StepOnePlus™ System Software (Applied Biosystems, Foster City, CA, USA) and ExpressionSuite Software (Applied Biosystems, Foster City, CA, USA) for inter-plate normalization. Relative Quantification (RQ) values (2^−ΔΔCt^, where Ct is the threshold cycle) of the target genes relative to their own endogenous control were obtained.

### 4.7. Statistical Analysis

Data are presented as mean with standard error of the mean (SEM) or standard deviation (SD) where appropriate. All data were tested for normality using the Shapiro-Wilk test. Non-normal biological data including glucocorticoid concentrations and relative quantification data were log transformed for analysis. Differences in demographics, psychiatric rating scales, and biological measures between depressed patients and healthy controls were assessed using a Mann-Whitney U test for independent sample comparisons between depressed and controls. A Pearson’s Chi-squared test was used to compare categorical variables between groups.

Correlational analysis was carried out using Spearman’s rho statistics on log transformed data. Psychiatric rating scale scores were correlated to cortisol parameters within the MDD group, and PSQI scores were correlated to cortisol parameters among the entire study population. Correlational analysis between relative quantification of *HSD11β-1* and cortisol parameters was carried out in the whole population, while correlational analysis of whole blood mRNA levels of inflammatory markers with cortisol parameters was conducted in the MDD group. All statistical analyses were considered significant when *p* ≤ 0.05. All data were analyzed using SPSS (Version 16, Armonk, NY, USA).

Graphs and statistics were generated using GraphPad Prism Software Version 5.00 (GraphPad Software, Inc., La Jolla, CA, USA). Raw values rather than log transformed values are depicted in figures.

## 5. Conclusions

Increased wakening cortisol and reduced CAR reactivity were found in depressed patients, which is consistent with previous research. This study provides strong evidence of HPA axis dysregulation in MDD primarily driven by elevated wakening cortisol concentrations. There were no differences in salivary cortisone concentrations or whole blood mRNA expression of *HSD11β-1* between groups. The data also support the hypothesis that increased inflammation occurs in tandem with the altered CAR in depression, indicating that glucocorticoid receptor downregulation may be responsible for both poor inhibition of the HPA axis and lack of suppression of the immune response in MDD patients.

## Figures and Tables

**Table 1:** Demographic data for patients with Major Depressive Disorder (MDD) and healthy controls for whom salivary cortisol and cortisone was measured by liquid chromatography-mass spectrometry (LC-MS).

Variable	Patients (*n* = 57)	Controls (*n* = 40)	Statistics (*p*-Value)
Age (years)	28.26 (8.41)	27.48 (5.61)	Z= −0.018, *p* = 0.985
Gender (female/male)	37/20	27/13	χ^2^ = 0.070, *p* = 0.831
BMI	24.96 (6.17)	22.81 (3.25)	Z = −1.459, *p* = 0.145
Smoking (Yes/No)	23/34	10/30	χ^2^ = 2.468, *p* = 0.133
ISCED Education	3.54 (1.54)	6.30 (1.45)	Z = −6.194, *p* < 0.001 ***
HAM-D	22.81 (5.14)	2.92 (2.80)	Z = −8.193, *p* < 0.001 ***
CES-D	39.04 (10.09)	6.85 (6.60)	Z = −8.123, *p* < 0.001 ***
PSQI	13.58 (3.36)	4.13 (2.70)	Z = −7.786, *p* < 0.001 ***
CTQ Global Score	44.50 (17.20)	30.43 (7.67)	Z = −3.999, *p* < 0.001 ***
Early Life Adversity (Yes/No)	35/22	8/32	χ^2^ = 19.136, *p* < 0.001 ***
Antidepressant use (Yes/No)	32/25	0/40	χ^2^ = 33.511, *p* < 0.001 ***

Data expressed as mean with SD in parentheses. Statistical analysis was performed using a Mann Whitney U test (Age, body mass index (BMI), Education, Hamilton Depression rating scale (HAM-D), Epidemiological Studies–Depression scale (CES-D), Pittsburgh Sleep Quality Index (PSQI), Childhood Trauma Questionnaire (CTQ)) and Chi squared (χ^2^) test (Gender, Smoking, Early Life Adversity (ELA), Antidepressant use). *** *p* ≤ 0.001 vs. control.

**Table 2:** Raw cortisol data for MDD patients and healthy controls.

Cortisol Measurement	Patients (*n* = 57)	Controls (*n* = 40)	Statistics (*p*-Value)
Cortisol T0	10.92 (1.34)	7.54 (0.64)	Z = −2.275, *p* = 0.023 *
Cortisol T30	12.93 (1.03)	11.00 (0.91)	Z = −1.156, *p* = 0.247
Cortisol T60	9.58 (1.13)	9.15 (0.85)	Z = −0.187, *p* = 0.852
Average AM Cortisol (nM)	11.46 (1.01)	9.09 (0.58)	Z = −1.300, *p* = 0.194
Cortisol T720	1.97 (0.29)	2.07 (0.65)	Z = −0.801, *p* = 0.423
Cortisol T750	2.12 (0.57)	1.96 (0.53)	Z = −0.132, *p* = 0.895
Average PM Cortisol (nM)	2.36 (0.55)	2.45 (0.68)	Z = −0.658, *p* = 0.510

Data are expressed as mean cortisol in nM with SEM in parentheses. Statistical analysis performed using a Mann Whitney-U test on log transformed cortisol data. AM = morning; PM = evening. * *p* ≤ 0.05 vs. control.

**Table 3:** Derived cortisol awakening response (CAR) parameters for MDD patients and healthy controls.

CAR Parameter	Patients (*n* = 57)	Controls (*n* = 41)	Statistics (*p*-Value)
AUCg	681.96 (58.88)	569.29 (38.95)	Z = −0.367, *p* = 0.367
AUCi	28.38 (63.97)	128.78 (41.76)	Z = −1.295, *p* = 0.195
Peak	15.41 (1.36)	12.68 (0.91)	Z = −1.120, *p* = 0.263
Reactivity	−1.71 (1.41)	1.37 (1.00)	Z = −1.962, *p* = 0.050 *
Intercept	12.46 (1.24)	8.41 (0.61)	Z = −2.045, *p* = 0.041 *
Slope	−0.039 (0.02)	0.023 (0.02)	Z = −1.977, *p* = 0.048 *

Data are expressed as mean with SEM in parentheses. Data expressed as mean with SEM in parentheses. Statistical analysis was performed using a Mann Whitney-U test on log transformed data. AUGg = area under the curve with respect to ground; AUCi = area under the curve with respect to increase. * *p* ≤ 0.05 vs. control.

**Table 4:** Raw cortisone data for patients with MDD and healthy controls.

Cortisone Measurement	Patients (*n* = 57)	Controls (*n* = 41)	Statistics (*p*-Value)
Cortisone T0	31.07 (2.03)	27.59 (1.76)	Z = −1.045, *p* = 0.296
Cortisone T30	39.85 (2.28)	33.99 (1.76)	Z = −1.436, *p* = 0.151
Cortisone T60	32.19 (2.22)	30.63 (1.54)	Z = −0.155, *p* = 0.877
Cortisone T720	10.93 (1.28)	9.72 (1.33)	Z = −0.459, *p* = 0.646
Cortisone T750	8.60 (1.00)	8.83 (1.14)	Z = −0.579, *p* = 0.562

Data are expressed as mean cortisone in nM with SEM in parentheses. Statistical analysis was performed using a Mann Whitney-U test on log transformed cortisone data.

**Table 5:** Cortisol awakening response parameter calculations.

Parameter	Definition	Formula
Diurnal variation	Change in cortisol concentration between morning time points and evening time points	=PM average − AM average
AUCg	Area under the curve with respect to ground	={[(T30 cortisol value + T0 cortisol value)/2] × time difference} + {[(T60 cortisol value + T30 cortisol value)/2] × time difference}
AUCi	Area under the curve with respect to increase	=AUC g − [T0 cortisol value × (total time between first and last measurement)]
Peak	Highest concentration of cortisol recorded	=Maximum cortisol value
Reactivity	Change in cortisol between first and last morning measurement	=T60 value − T0 value
Slope	Slope of the regression line fitted through raw morning cortisol data	=correlation coefficient r × [SD of AM cortisol values/SD of AM time values]
Intercept	Intercept of the regression line fitted through raw cortisol data	=the mean of cortisol values − (slope × mean of 3 AM cortisol values)

## Abbreviations

MDD	Major Depressive Disorder
CAR	cortisol awakening response
HPA	hypothalamic-pituitary-adrenal
HAM-D	Hamilton Depression rating scale
CES-D	Center for Epidemiology Scale–Depression
PSQI	Pittsburgh Sleep Quality Index
CTQ	Childhood Trauma Questionnaire
IL	interleukin
TNF	tumor necrosis factor
IFN	interferon
RQ	relative quantification
LC-MS	liquid chromatography-mass spectrometry
PSQI	qualitative polymerase chain reaction
AUC	area under curve
SSRI	selective serotonin reuptake inhibitor
mRNA	messenger ribonucleic acid
SD	standard deviation
SEM	standard error of the mean
